# Stream microbial diversity in response to environmental changes: review and synthesis of existing research

**DOI:** 10.3389/fmicb.2015.00454

**Published:** 2015-05-18

**Authors:** Lydia H. Zeglin

**Affiliations:** Division of Biology, Kansas State UniversityManhattan, KS, USA

**Keywords:** ecosystem structure and function, lotic ecosystems, microbial diversity, rivers, streams

## Abstract

The importance of microbial activity to ecosystem function in aquatic ecosystems is well established, but microbial diversity has been less frequently addressed. This review and synthesis of 100s of published studies on stream microbial diversity shows that factors known to drive ecosystem processes, such as nutrient availability, hydrology, metal contamination, contrasting land-use and temperature, also cause heterogeneity in bacterial diversity. Temporal heterogeneity in stream bacterial diversity was frequently observed, reflecting the dynamic nature of both stream ecosystems and microbial community composition. However, within-stream spatial differences in stream bacterial diversity were more commonly observed, driven specifically by different organic matter (OM) compartments. Bacterial phyla showed similar patterns in relative abundance with regard to compartment type across different streams. For example, surface water contained the highest relative abundance of Actinobacteria, while epilithon contained the highest relative abundance of Cyanobacteria and Bacteroidetes. This suggests that contrasting physical and/or nutritional habitats characterized by different stream OM compartment types may select for certain bacterial lineages. When comparing the prevalence of physicochemical effects on stream bacterial diversity, effects of changing metal concentrations were most, while effects of differences in nutrient concentrations were least frequently observed. This may indicate that although changing nutrient concentrations do tend to affect microbial diversity, other environmental factors are more likely to alter stream microbial diversity and function. The common observation of connections between ecosystem process drivers and microbial diversity suggests that microbial taxonomic turnover could mediate ecosystem-scale responses to changing environmental conditions, including both microbial habitat distribution and physicochemical factors.

## Introduction

Aquatic microbial diversity is well understood to be a key component of aquatic ecosystem functioning (Nold and Zwart, 1998; Cotner and Biddanda, 2002; Gessner et al., 2010), and major advances toward linking microbial diversity with ecosystem function have been made in aquatic systems (Horner-Devine et al., 2003; Smith, 2007; Singer et al., 2010; Comte et al., 2013). While the responsiveness of microbial diversity to environmental variability is an ongoing topic of inquiry (Finlay, 2002; Allison and Martiny, 2008; Langenheder et al., 2012; Shade et al., 2012), it is clear that global environmental change poses many potential threats to the structure and function of all aquatic ecosystems (Malmqvist and Rundle, 2002; Dudgeon et al., 2006), and that changing environmental factors at the watershed scale directly impact the biological function of lotic ecosystems (Likens et al., 1970; Hynes, 1975; Mulholland et al., 2008; Palmer and Febria, 2012). However, a recent survey of microbial diversity studies in aquatic habitats showed that microbial diversity in lotic environments is less commonly studied than in marine and lake ecosystems, and that impacted systems are less commonly studied than unimpacted systems (Zinger et al., 2012). Given the importance of microbial processes to lotic ecosystem function, and the microbial genetic diversity that contains the information supporting those functions as well as the potential for resilience under environmental changes, it is critical to study stream microbial diversity in the context of shifting environmental drivers.

Streams and rivers are hotspots of microbially mediated carbon (C) and nutrient processing within landscapes (Hynes, 1975; Fisher et al., 1998; McClain et al., 2003). Microbial activity drives organic matter (OM) decomposition, whole-stream respiration and C flow to higher trophic levels (Lindeman, 1942; Meyer, 1994; Hall and Meyer, 1998; Hieber and Gessner, 2002; Tank et al., 2010). Thus, microbial processes are at the center of the conceptual model of OM processing and food web structure through the river continuum (Vannote et al., 1980; Minshall et al., 1985). Also, microbial nitrification, denitrification, and heterotrophic nitrogen (N) uptake in small streams affects downstream water quality (Peterson et al., 2001; Mulholland et al., 2008; Valett et al., 2008). There is a strong history and impact of studying microbial processes within and among stream and river ecosystems, yet molecular methods, which enable the study of microbial diversity within the context of ecosystem function, have not been widely utilized in lotic ecosystems (Findlay, 2010).

Studies on bacterial and fungal diversity in lotic ecosystems have been historically more associated with the research themes of microbial transport and leaf litter decomposition, respectively. Stream fungal diversity research has traditionally been rooted in an ecological context, investigating the patterns and mechanisms of aquatic hypomycete succession that occur on leaf biofilms concomitant with the progression of leaf decomposition (Suberkropp and Klug, 1976; Suberkropp et al., 1976; Webster and Descals, 1981; Bärlocher, 1982; Gessner et al., 1993). Early stream bacterial diversity research consisted of culture-for-diversity assessments of bacterial loads in the water column, resulting in predominantly *Pseudomonas* sp. isolates with variation in diversity correlated most strongly with water temperature, storm events and sunshine (Bell et al., 1982a,b). These studies relied upon microscopy for identification of fungal conidia following sporulation or substrate utilization profiling of bacterial isolates. Bacterial diversity was particularly difficult to define, beyond differentiating gram-negative from gram-positive cells (Geesey et al., 1977) or conducting plate counts on selective media (Milner and Goulder, 1984), before the availability of molecular tools.

The application of molecular techniques to measure stream microbial diversity produced new insights. Genetic markers within each “*Pseudomonas”* sp. isolate differed among isolates derived from the same stream, and between stream- and soil-derived isolates (McArthur et al., 1992); somewhat analogously, the distribution of selected loci differed among *Tetrachaetum elegans* monosporic isolates from different leaf species within the same stream (Charcosset and Gardes, 1999). Also, the genetic composition of the entire microbiota found on fine particulate OM was more similar than expected given differences in water chemistry among the streams sampled (Sinsabaugh et al., 1992). Following the establishment of the ribosomal rRNA gene as a conserved marker of taxonomic lineage (Pace, 1997), studies on water column biota began to resolve longitudinal patterns in microbial diversity (Crump et al., 1999; Crump and Baross, 2000), and efforts to integrate molecular and traditional tools in studies of fungal diversity were mounted (Nikolcheva et al., 2003; Nikolcheva and Bärlocher, 2005). In the 21st century, studies of molecular microbial diversity in lotic ecosystems are increasing to an extent that a review and synthesis of progress on the topic is needed.

Because microbial processes in stream and river ecosystems are variable in space and time in response to differences in nutrient availability (Dodds et al., 2000), temperature (Boyero et al., 2011), OM quality or quantity (Gessner and Chauvet, 1994), hydrological factors (Valett et al., 1997), or land use (Mulholland et al., 2008), a reasonable initial prediction is that microbial diversity might also respond to changes in these environmental variables. In fact, many recent studies of molecular microbial diversity in streams have been initiated based on this rationale. Among these case studies, there are many examples of variation in microbial diversity in response to environmental variability, many examples of microbial diversity showing no response to the predicted driver, and many examples of microbial diversity changing with one environmental factor, but not another, in the same study. This leads to a large degree of uncertainty in the extent to which lotic microbiota are sensitive or resistant to environmental perturbation (Allison and Martiny, 2008; Shade et al., 2012). If microbial functions are phylogentically conserved to any extent, there should be some predictability to the changes in microbial diversity along environmental gradients in space and time (Phillippot et al., 2010). If functional redundancy among microbial taxa or physiological flexibility due to functional diversity within microbial taxa is high, microbial diversity will be more static in the face of environmental fluctuation. One literature review cannot tease apart these complex mechanisms; however, it can attempt to identify which environmental factors are demonstrated to be more or less likely to affect microbial diversity. Any pattern can inform hypotheses regarding the sensitivity or resilience of diverse aquatic microbiota to the multiple categories of current environmental threats to aquatic ecosystems (Malmqvist and Rundle, 2002).

With this goal in mind, I gathered published papers on microbial diversity in streams and rivers for a focused review. After collecting papers, I recorded the findings of each in a categorical manner: I noted the significance or lack thereof for each comparison within each study, differentiated among the effects of defined environmental drivers and contrasting levels of spatiotemporal variation, tallied the frequency of studies using various methodologies, and harvested coarse taxonomic data from relevant studies. The result is a synthesis that is a step beyond a traditional review, but not as quantitatively rigorous as a true meta-analysis. There was a great deal of evidence for sensitivity of lotic microbial communities to environmental variation, and somewhat surprising implications regarding the relative influence of physical versus geochemical factors on microbial diversity.

## Methods

To collect as many published, peer-reviewed studies on stream microbial diversity as possible, I searched the Web of Science database using the parameters TS = (“stream” OR “river” OR “lotic”) AND TS = (microb^∗^ OR bacteria^∗^ OR fung^∗^) AND TS = “diversity” for all full years available (through 2013). I screened search results and kept all papers that (i) reported microbial taxonomic diversity including data collected using molecular, microscopy, and “culture for diversity” methods and (ii) evaluated the effect of some environmental variable on microbial diversity. I accepted papers that reported diversity as richness, as a diversity index incorporating some estimate of relative abundance of taxa, or as the relative abundance of measured taxa (community structure). These search parameters may not catch some relevant studies, however, the resulting synthesis of 294 papers is to my knowledge the most comprehensive to date.

These studies were categorized based on (i) the microbial group of interest (fungi, bacteria, protozoa, archaea, stramenopiles); (ii) the methodology used (microscopy, denaturing gradient gel electrophoresis (DGGE), terminal restriction fragment length polymorphism (T-RFLP), sequencing of genes from clone libraries, sequencing of genes from next-generation technology libraries, fluorescent *in situ* hybridization (FISH), other molecular methods and other non-molecular methods; and (iii) the environmental driver(s) evaluated by the study (temporal variation, among-stream variation, longitudinal variation, differences in nutrient concentrations, differences in OM quantity or quality, OM compartment type, hydrologic variation, differences in metals concentrations, differences in surrounding land-use, and differences in temperature, **Table 1**). All studies were also categorized by the OM compartment sampled coarse particular organic matter (CPOM, leaves or wood), water column, epilithon (biofilm attached to any hard surface), streambed sediment, hyporheic biofilm or water, and other [including, e.g., foam, fine benthic organic matter (FBOM)], and by the scale of investigation (within-stream comparison, among-stream comparison, or among-region comparison). Many studies included multiple experiments or comparisons, or comparisons that could be classified under multiple categories (e.g., a study evaluating the effect of a wastewater treatment plant on bacterioplankton diversity was categorized as a longitudinal comparison and as a comparison of nutrient concentration effects).

**Table 1:** Categories of environmental variation evaluated for effects on stream microbial diversity.

Category	Definition/Variables included
**Spatiotemporal variation**
Temporal variation (*n* = 101)	Samples collected at multiple time points
Among-stream variation (*n* = 82)	Samples collected at different streams
Longitudinal variation (*n* = 70)	Samples collected at different sites from up-to-downstream
Compartment type (*n* = 38)	At one site within a stream, samples collected from different OM/surface types (including rocks, coarse particular organic matter (CPOM), benthic surface sediment, subsurface sediment, or no surface i.e., water column)
**Variation in defined environmental drivers**
Nutrient concentrations (*n* = 56)	Variation in surface water nutrient concentrations; nutrient = any form of N or P, or C:N, C:P, or N:P stoichiometry
Organic matter (OM) quality/quantity (*n* = 52)	Variation in surface water DOC concentrations, particulate OM stock, or substrate quality (e.g., different species of leaf litter)
Hydrological variation (*n* = 32)	Variation in stream flow, hydrological regime, or before/after a defined flooding or drying event
Metals effects (*n* = 31)	Variation in soluble metals concentrations (e.g., Al, Cd, Cu, Fe, Mn, Pb, Zn), or generalized acid mine drainage effects
Land-use (*n* = 28)	Variation in riparian or watershed land-use (e.g., agricultural, urban, undeveloped)
Temperature (*n* = 23)	Variation in water temperature

*Studies were classified into categories of spatiotemporal variation or variation in environmental drivers based on the sampling strategy employed in each study, with number (n) of studies noted for each category*.

It is not valid to compare the values of derived diversity metrics or the abundance of microorganisms based on data collected with different methodologies and taxonomic resolutions, so a fully quantitative metaanalysis, using a response index, was not possible. However, it is valid to accept significant results of a study as informative, no matter the data type. For example, a meta-analysis of heterogeneity in soil microbial communities showed greater-than-random spatial similarity no matter the technique used to measure diversity, but the magnitude of heterogeneity detected was greater if a lower-resolution taxonomic definition was utilized (Horner-Devine et al., 2007). Therefore, for this study, a qualitative comparative approach was used: Each comparison of environmental variability on stream microbial diversity was categorized as “significant” or “non-significant,” based on the criteria used in the publication, and the distribution of significant results was compared among all studies. With this approach, a semi-quantitative evaluation of the most and least commonly observed effects of environmental drivers on stream microbial diversity was possible.

Some studies in the database reported the 16S rRNA gene sequence abundance of microbial taxa. While individual datasets had varied sequencing depths, particularly the clone library versus next-generation sequencing studies, the most abundant microbial populations should be the most commonly sequenced using any method. In the interest of extracting as much information as possible from the database, I harvested relative abundances of dominant phyla and subphyla in bacterial 16S rRNA gene libraries from the 29 studies that reported relative abundance in addition to diversity/heterogeneity summary metrics. This data extraction included neither studies that reported sequence information for only a subset of sequences gathered (thus skewing relative abundances higher), nor FISH data, which, while based on the 16S rRNA gene sequence, tend to not quantify bacterial phyla such as Acidobacteria, Cyanobacteria, or Verrucomicrobia. While differences in extraction protocol, sequencing primers, sequencing depth, and other technical particulars introduce potentially large sources of non-environmental variation to this analysis, signals that rise above the noise must be particularly strong. I tested the hypothesis that OM compartment type affected bacterial community composition across studies with a one-way analysis of variance (ANOVA) and Bonferonni *post hoc* comparisons using R Commander (Fox, 2005; R Core Development Team, 2010).

## Results

**FIGURE 1:**
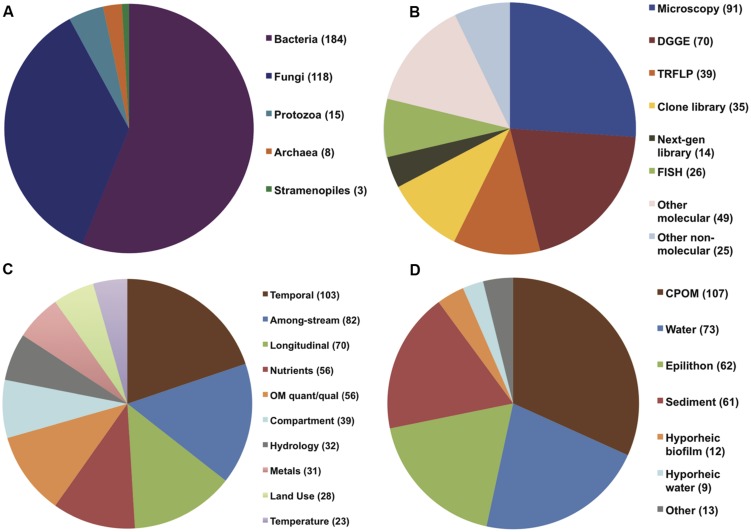
**Pie charts showing distribution of (A) organism of study, (B) methodology used in study, (C) environmental drivers, and (D) compartment of study among all papers (294) included in the analysis**.

**FIGURE 2:**
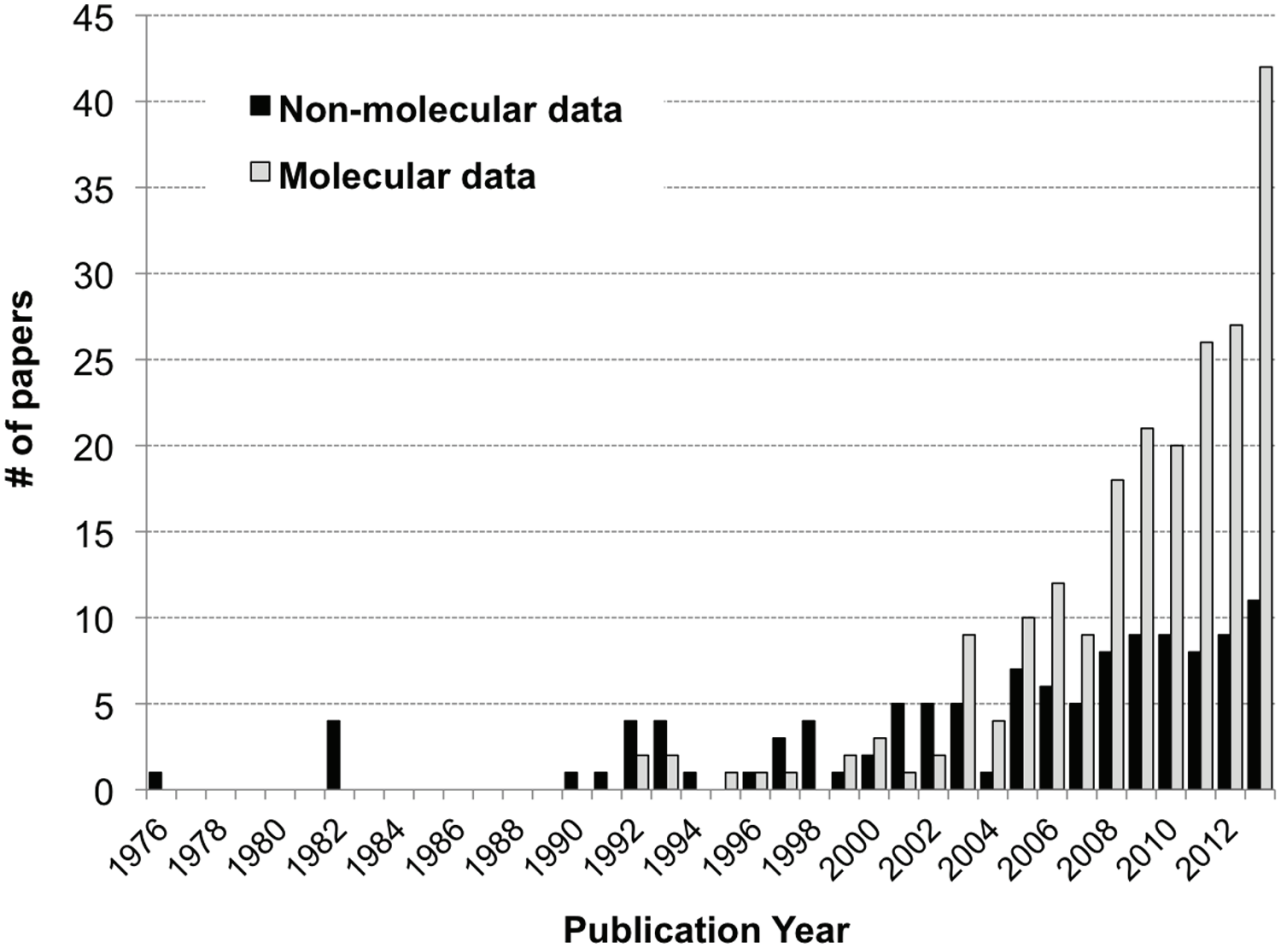
**Histogram of the number of papers reporting stream microbial diversity from 1992 to 2013: papers using molecular methods as gray bars, non-molecular methods as black bars**.

A total of 294 papers published between 1976 and 2013 were included in the analysis; these papers contained 520 comparisons of the effects of spatiotemporal heterogeneity or defined environmental drivers on microbial community composition in streams (Appendix). The majority of papers examined bacterial communities (56%), many examined fungal communities (36%), and the remainder examined archaeal, protozoan, or stramenopile communities (**Figure 1**). Many fungal studies utilized a definition of taxonomic identity based on conidial morphology, making microscopy the most common methodology in the database (26% of studies), followed by the “community fingerprint” techniques of DGGE and T-RFLP (20 and 11% of studies, respectively) and sequencing of ribosomal genes from clone libraries (10%; **Figure 1**). Only 4% of studies utilized next-generation sequencing, all published in 2012 or 2013; this reflects a more rapid increase in papers using molecular methods in the past decade (**Figure 2**). In total, almost half of the comparisons measured spatiotemporal variation in microbial community composition (temporal, 20%; among-stream, 16%; longitudinal, 14%), and the most commonly evaluated environmental drivers were nutrient concentration (11%) and OM quality or quantity (11%; **Figure 1**). Finally, among compartment types studied, CPOM was best represented (32%, primarily fungal communities), followed by the water column (22%, primarily bacterioplankton), epilithic biofilms (18%), and benthic sediment (18%; **Figure 1**).

**FIGURE 3:**
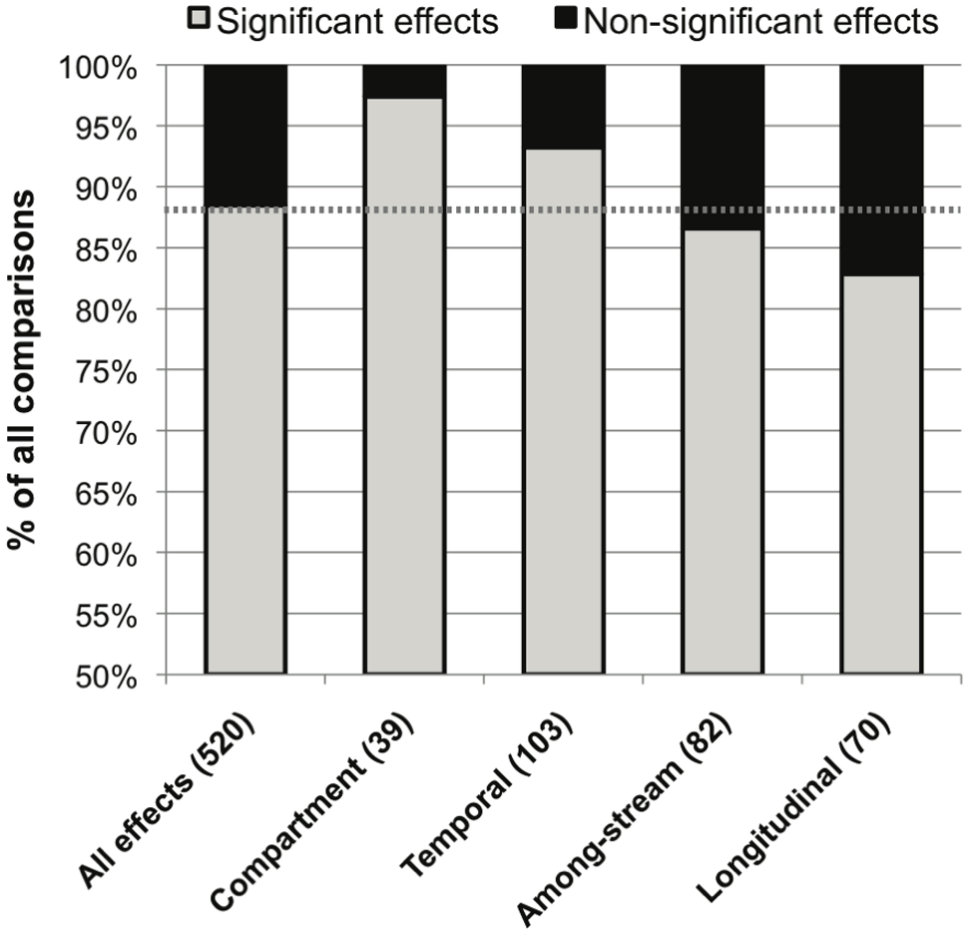
**Distribution of significant (in gray) and non-significant (in black) reported effects of categories of spatiotemporal variation on stream microbial diversity, with number of included studies noted for each category along the x-axis, and the percentage of significant effects for all comparisons combined noted as a dashed line for reference**.

The overall distribution of published significant versus non-significant effects of environmental variation on stream microbial community composition was 88.5% versus 11.5%. The most common source of spatio-temporal heterogeneity was within-stream heterogeneity among different OM compartments (97% significance among published effects), followed by within-stream temporal, among-stream, and within-stream longitudinal heterogeneity (93, 87, and 83% significant effects, respectively; **Figure 3**).

**FIGURE 4:**
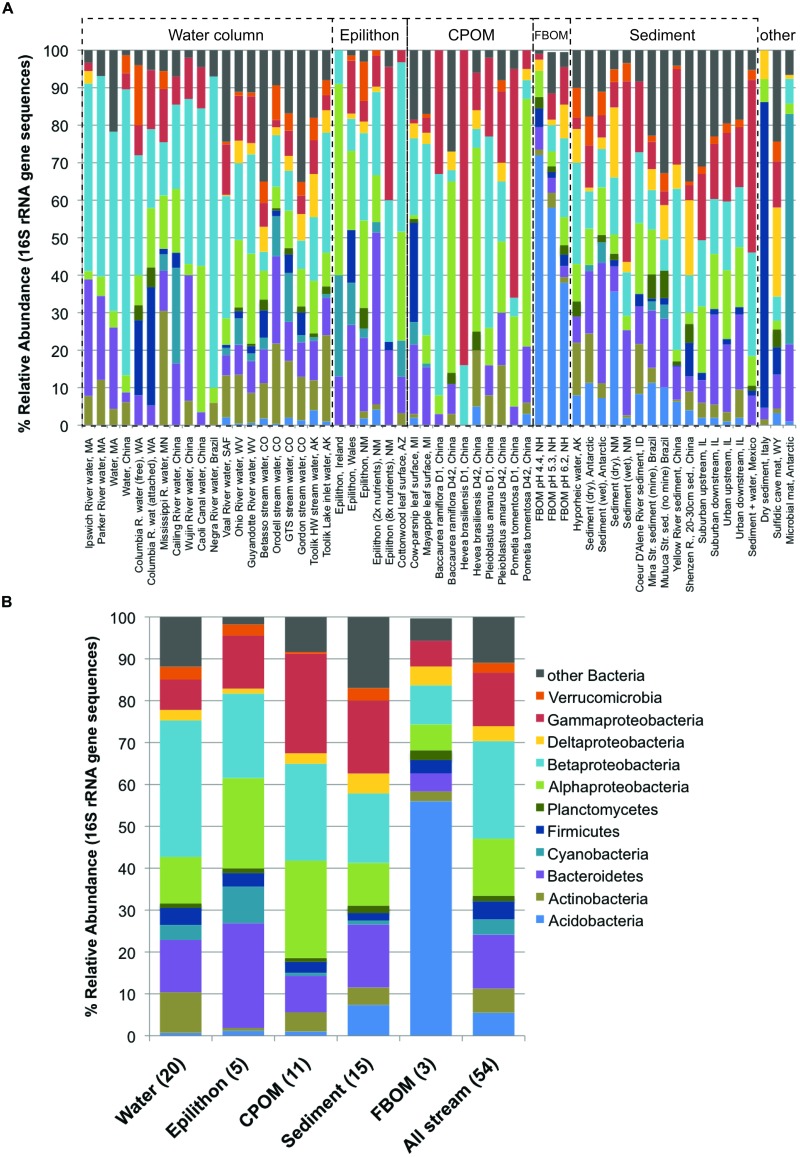
**Relative abundance of major bacterial phyla and subphyla (based on 16S rRNA gene sequence libraries, available from 29 papers) for all available defined compartments: (A) all samples with habitats and locations noted along the *x*-axis and compartment type bracketed by dashed lines, noted above each group; (B) average community composition for each compartment**.

Because a good number of studies reported the dominant bacterial phyla and subphyla in 16S rRNA gene sequence libraries from defined sample types (either clone libraries, 21 studies; or next-generation sequencing, eight studies), it was possible to evaluate which taxonomic groups varied in relative abundance among compartments (**Figure 4**). The results of ANOVA *post hoc* comparisons showed significant among-compartment differentiation in a number of phyla, including the Acidobacteria, Actinobacteria, Bacteroidetes, Cyanobacteria, and other Bacteria (**Table 2**). Among different stream compartments, water column samples contained the highest relative abundance of Actinobacteria, epilithon samples contained the highest relative abundance of Bacteroidetes and Cyanobacteria, FBOM samples contained the highest relative abundance of Acidobacteria, and sediment samples contained the second highest relative abundance of Acidobacteria and the highest relative abundance of other Bacterial sequences.

**Table 2:** Relative abundance [%, (1 SE)] of bacterial phyla and subphyla (rows) in stream compartments (columns) including analysis of variance (ANOVA) results for among-compartment comparisons: omnibus results in first column, and significant multiple comparisons groups (Bonferonni *post hoc* test, α = 0.05) in lower case superscripts (a-d).

	ANOVA results (*F, P*)	Water column	Epilithon	CPOM	Fine benthic organic matter (FBOM)	Sediment
Acidobacteria	60.9, <0.0001	0.69^a^ (0.24)	1.20^ab^ (0.82)	0.91^ab^ (0.51)	56.0^d^ (9.9)	7.40^c^ (2.2)
Actinobacteria	3.15, 0.022	9.70^b^ (1.8)	0.65^a^ (0.40)	4.48^ab^ (1.8)	2.33^ab^ (1.3)	4.81^ab^ (0.83)
Bacteroidetes	4.02, 0.007	12.5^a^ (9.3)	25.0^b^ (13)	8.79^a^ (6.4)	4.33^a^ (1.5)	14.5^ab^ (8.1)
Cyanobacteria	2.46, 0.058	3.57^ab^ (1.4)	8.69^b^ (5.6)	3.25^ab^ (0.98)	0.00^a^ (0)	0.88^ab^ (0.44)
Firmicutes	0.319, 0.864	4.09 (1.8)	3.27 (2.7)	2.41 (2.4)	3.17 (1.0)	1.68 (0.58)
Planctomycetes	0.852, 0.499	1.03 (0.34)	1.10 (1.1)	0.82 (0.50)	2.33 (0.44)	1.91 (0.65)
Alphaproteobacteria	2.74, 0.039	11.1 (2.0)	21.6 (8.4)	23.8 (6.8)	6.17 (1.1)	10.2 (1.6)
Betaproteobacteria	2.26, 0.076	32.6 (2.7)	20.2 (5.4)	25.2 (6.6)	9.33 (6.2)	17.3 (2.4)
Deltaproteobacteria	1.27, 0.296	2.50 (0.51)	1.17 (0.57)	2.27 (0.69)	4.50 (2.3)	5.04 (1.7)
Gammaproteobacteria	1.96, 0.115	7.26 (1.1)	12.7 (6.2)	21.9 (8.3)	6.17 (2.5)	16.5 (3.6)
Verrucomicrobia	2.10, 0.095	3.07 (0.92)	2.64 (1.97)	0.36 (0.28)	0.00 (0)	3.30 (0.64)
Other bacteria	3.03, 0.026	11.9^ab^ (2.3)	1.78^a^ (0.87)	7.59^ab^ (2.8)	5.33^ab^ (3.0)	16.5^b^ (2.8)

**FIGURE 5:**
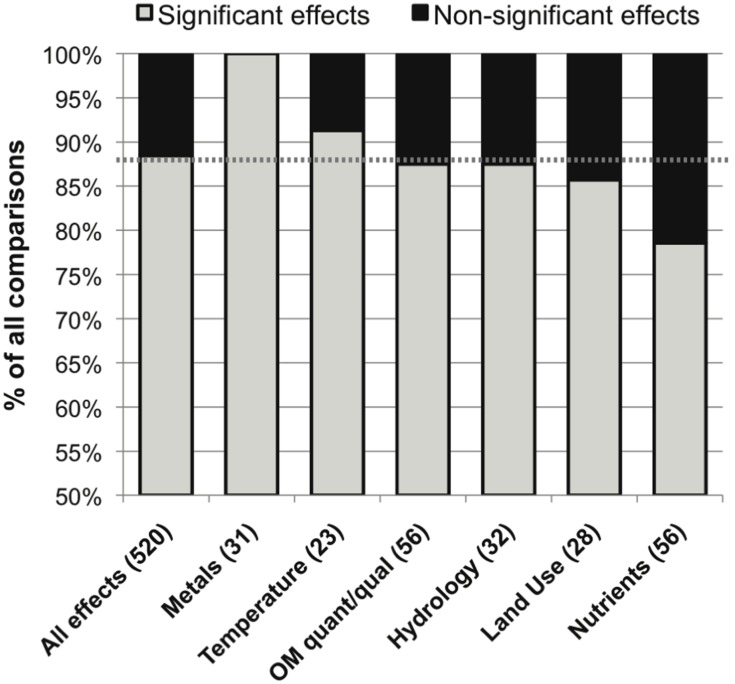
**Distribution of significant (in gray) and non-significant (in black) reported effects of categories of variation in defined environmental drivers on stream microbial diversity, with number of included studies noted for each category along the x-axis, and the percentage of significant effects for all comparisons combined noted as a dashed line for reference**.

The category of defined environmental drivers with the most commonly significant effect was metals (100%), followed by temperature, OM quantity or quality and hydrology, land use and nutrient concentrations (91, 88, 88, 86 and 79% significant effects, respectively; **Figure 5**). The studies evaluating metals effects did not report widely comparable taxonomic data (these included a mix of fungal microscopic, fungal sequencing, bacterial sequencing, and non-sequencing data), so it was not possible to perform an evaluation of metals effects on microbial community composition.

**FIGURE 6:**
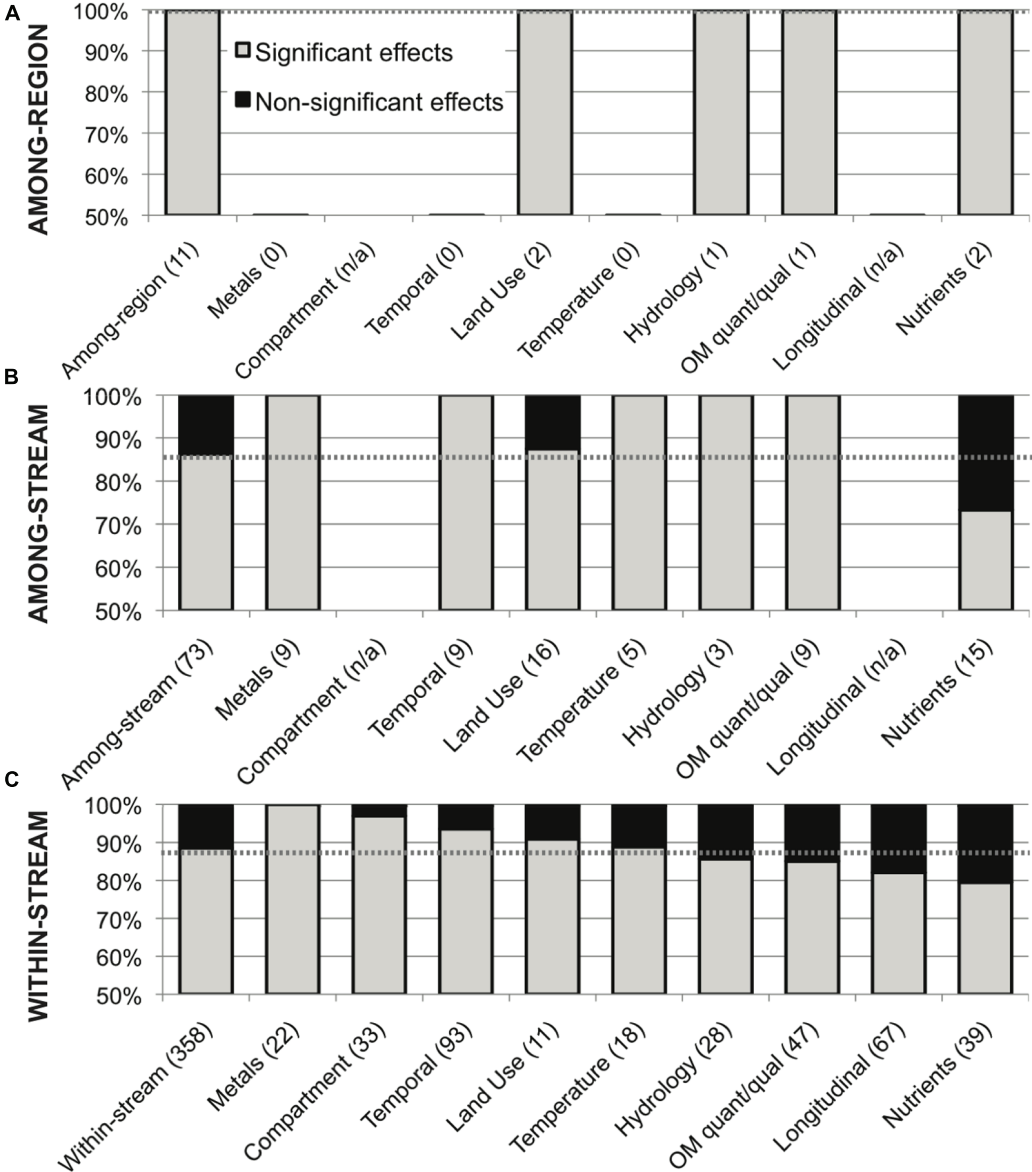
**Distribution of significant (in gray) and non-significant (in black) reported effects of categories of variation in all environmental drivers on stream microbial diversity, for (A) among-region comparisons, (B) among-stream comparisons and (C) within-stream comparisons; with number of included studies noted for each category along the x-axis, and the percentage of significant effects for all comparisons combined noted as a dashed line for reference, for each scale**.

This meta-analysis of environmental variation in stream microbial diversity was dominated by within-stream studies (358 comparisons, 88.5% significant); to contrast with other study scales, the 73 among-stream comparisons showed a total distribution of 86% significant effects, with 100% significance within all categories of variation except land use and nutrient concentrations (88 and 73% significant effects, respectively), and the 11 among-region comparisons showed 100% significance within all categories of variation (**Figure 6**).

## Discussion

The results of this data synthesis reflected a high incidence of microbial diversity responses to environmental variation in stream and river ecosystems. Among spatiotemporal factors, within-stream compartment differences and temporal differences were most common, while longitundinal differences were least common. Among defined drivers, “metals” effects were ubiquitous and land-use and nutrients effects were least common. Overall, lotic microbial communities are quite sensitive to environmental changes, but their functional redundancy may be greater in relation to certain environmental variables than others.

The relative distribution of significant and non-significant effects of different categories of environmental drivers on lotic microbial diversity provides an informative synthesis of the current state of the literature. However, the high number of significant comparisons – 88.5% – raises some concern of a literature bias favoring the publication of significant results. This is an established concern in the interpretation of metaanalyses, and can result from a lack of enthusiasm by authors or reviewers to publish a study with “no effect” (Rosenthal, 1979; Hedges, 1992). This phenomenon may occur to some extent, which is unfortunate since non-significant results are important information within the context of the broader state of knowledge on a subject, as this synthesis demonstrates. On the other hand, investigators form an hypothesis based on a justified rationale; in this case, the expectation that an environmental factor known to affect microbial processes might also affect microbial diversity (Allison and Martiny, 2008). The high prevalence of significant variation in lotic microbial diversity in response to all evaluated drivers is most likely a consequence of widespread microbial sensitivity to environmental change (Shade et al., 2012).

### Spatiotemporal Heterogeneity

Microbes may be particularly sensitive to changes in their environment due to their small size and rapid growth rates. This high microbial turnover potential is reflected as a higher prevalence of temporal variation (93%) than longitudinal (83%) or among-stream (86%) variation in lotic microbiota (**Figures 3** and **6**). This temporal variation includes seasonal changes (Feris et al., 2003b; Olapade and Leff, 2004; Crump and Hobbie, 2005; Hullar et al., 2006), successional turnover on the order of days to weeks (Gessner et al., 1993; Lyautey et al., 2005; Wey et al., 2012; Wymore et al., 2013), and shifts with transient or less predictable durations directly associated with hydrological fluctuations (Battin et al., 2001; Rees et al., 2006; Chiaramonte et al., 2013; Fazi et al., 2013). These temporal changes can be correlated with multiple defined environmental drivers: Most notably, seasonal changes in water temperature, hydrology, OM quality and nutrient availability can be relatively predictable, and seasonal synchrony among years and streams in lotic bacterioplankton, epilithon, and sediment microbial diversity has been documented (Sutton and Findlay, 2003; Hullar et al., 2006; Crump et al., 2009). This is intriguing since it suggests that microbial community composition could be predictable based on environmental factors, as has been shown in lakes, estuaries, and oceans (Fuhrman et al., 2006; Ladau et al., 2013). However, predictability could be particularly challenging in stream and river ecosystems due to their variable hydrology and associated potential for cell dispersal via microbial transport (Bell et al., 1982a; Crump et al., 2012). Compared to other aquatic ecosystems, soils, and several other broad habitat types, stream ecosystems have the highest measured indices of temporal variability in microbial community composition (Portillo et al., 2012; Shade et al., 2013). Stream and river ecosystems, and their macrofauna, are characterized by temporal variability (Poff and Ward, 1989), and a high level of temporal turnover in microbial community composition may also be characteristic of lotic systems.

An even stronger and perhaps more surprising result was the widespread effect of compartment type on microbial diversity (**Figure 3**). Differences in stream microbiota between surface water, rock surfaces, leaves and wood, and streambed sediment – within the same stream – were more common than any other spatiotemporal effect. Only one study found similar microbial diversity between compartment types; this study took place in a contaminated stream where the high metals concentrations were hypothesized to limit fungal diversity (Sridhar et al., 2008). Bacterial community composition was remarkably consistent within compartment types in samples collected from a wide range of sites (**Figure 4**). The high relative abundance of Cyanobacteria and Bacteroidetes in epilithic biofilm makes sense, as autotrophic microbes such as Cyanobacteria are more competitive on an inorganic substrate, and Bacteroidetes are known to be characteristic of well-developed stream biofilms (Besemer et al., 2009). Stream and river sediments might contain more pore-scale heterogeneity and anaerobic microsites (Kemp and Dodds, 2001) that promote a greater abundance of narrower phyla or unknown taxa. It is less clear why Actinobacteria might be characteristic of surface waters or Acidobacteria might be characteristic of FBOM. This result does suggest, however, that characteristics of the within-stream physicochemical environment could select for specific groups of microorganisms, and that some physiological characteristics allowing successful colonization of different microbial habitats may be conserved at the phylum level (Phillippot et al., 2010).

The major physicochemical differences among lotic “microbial habitat” compartments are primarily OM type, which could select for microbes best suited to different categories of C processing, and surface type, which could select for microbes best suited to attachment and growth under certain conditions. The CPOM habitat consists of primarily complex polymeric OM, lignin, cellulose and lignocellulose, which can select for microbes that produce extracellular enzymes with oxidative and hydrolytic capabilities, such as fungi. Fine particulate OM habitat contains fragmented and processed OM with more surface area for bacterial colonization (Sinsabaugh et al., 1992). The sediment habitat may be the most heterogeneous in terms of OM type, containing a mixture of buried OM of all sizes and ages, and in terms of physical structure, with inorganic particles of widely varied texture and the potential to set up steep diversity and productivity gradients under saturated conditions (Ellis et al., 1998; Findlay et al., 2003). The epilithic habitat provides no OM at early stages in biofilm development, though mature biofilms contain primary producers that exude C compounds, and entrain particulate OM. Water column habitat supports both suspended and particle-associated bacterioplankton (Crump et al., 1999) and contains a combination of algal and exogenous C. In addition to the contrasting OM substrates characterizing these habitats, they present surfaces with different texture and area for microbial colonization: surface roughness and water flow conditions affect the trajectory and diversity of cells inhabiting biofilms and the exchange of cells and particles between biofilms and the surface water (Battin et al., 2003; Arnon et al., 2010; Singer et al., 2010). This mosaic of selective habitats within a stream (Pringle et al., 1988), combined with the strong mass effects of flowing waters (Crump et al., 2007, 2012), make lotic ecosystems prime test grounds for questions about microbial community assembly within a metacommunity context (Logue et al., 2011).

### Defined Drivers Effects

Specific drivers of environmental change had varied frequencies of significant influence on stream and river microbial diversity (**Figure 5**), with the exception of the always-significant effect of metals. As noted earlier, the only study that reported no significant differentiation among OM compartments was undertaken in metal-contaminated streams (**Figure 3**; Sridhar et al., 2008). This ubiquitous result reflects the acute toxic effects that metals can have on cells with no tolerance adaptations (Genter and Lehman, 2000). Also, large changes in pH and concentrations of electron donors and acceptors, as found in acid mine drainage streams, creates conditions favorable for very specific microbial metabolic functions and taxonomic groups (Niyogi et al., 2002; Kim et al., 2009; Yu et al., 2010). In soils, pH can vary widely and is correlated with broad differences in microbial community composition and function (Sinsabaugh et al., 2008; Lauber et al., 2009; Rousk et al., 2010); however, in lotic ecosystems the isolated effect of pH on microbial diversity has been studied less frequently and has varied effects on microbial diversity (Fierer et al., 2007; Simon et al., 2009). Field studies within the “metals” category were associated with current or historic mining or industrial activities in the watershed (Feris et al., 2003a; Ancion et al., 2010); however, none were associated with an effect of pH in isolation from metals contamination (Baudoin et al., 2008). This reflects the common covariance of multiple environmental stressors, such as metals and pH, associated with watershed mining activities (Palmer et al., 2010) and urbanization (Grimm et al., 2008), and highlights the dramatic impact that these factors may have in delimiting microbial niches.

In contrast, significant land-use and nutrients effects on lotic microbial diversity were least often observed (**Figures 5** and **6**). This may point to a relatively high level of functional diversity and redundancy regarding nutrient kinetics within natural microbial communities: cells may have strategies to operate under a range of nutrient concentrations (Findlay and Sinsabaugh, 2003), and taxa with contrasting nutrient affinities may often coexist (Martens-Habbena et al., 2009), possibly due to dormancy or low but persistent viability during suboptimal conditions (Jones and Lennon, 2010; Shade et al., 2014). In complement, other environmental drivers may have a stronger effect on limiting the competitive advantage of some microbial taxa than nutrient availability. Metals concentrations (Ancion et al., 2013), temperature (Sliva and Williams, 2005; Zhang et al., 2012), OM quantity or quality (Zoppini et al., 2010; Marano et al., 2011), hydrological factors (Sliva and Williams, 2005; Zoppini et al., 2010), or other site-specific factors (Oliveira and Goulder, 2006; Comte and del Giorgio, 2009; Perez et al., 2012; Washington et al., 2013) could cause stricter physiological limitations on cell success than nutrient availability. This functional redundancy and relative insensitivity of stream and river microbiota to changing nutrient concentrations helps to explain situations in which nutrient concentrations are better predictors of microbial function than microbial diversity (Baxter et al., 2012).

On the other hand, it is important to remember that, while least common, nutrient effects on lotic microbial diversity were significant in 79% of studies. These effects included a number of experiments that applied nutrient enrichment in the absence of other environmental variation (Sridhar and Bärlocher, 2000; Olapade and Leff, 2005; Artigas et al., 2008; Van Horn et al., 2011). Many environmental nutrient gradients include covariates, however. Wastewater treatment eﬄuent, which can cause shifts in river temperature, salinity and bacterial load, as well as increased nutrient concentration, was often influential on microbial diversity (Wakelin et al., 2008; Angel et al., 2010; Drury et al., 2013; Sonthiphand et al., 2013). Nutrient impacts on stream microbiota from land-use change are often accompanied by differences in riparian vegetation and cover (thus OM quality), temperature, geomorphology and hydrological connectivity, and organic pollutants (Findlay and Sinsabaugh, 2006; Dorigo et al., 2009; Villeneuve et al., 2011). Thus, it is critical to consider both the individual and interactive impacts of changes in nutrient concentrations and other environmental alterations on microbial diversity, functional redundancy, and integrated function. Future studies on lotic microbial nutrient sensitivity should strive to utilize experimental data, in addition to observational data, to make the largest strides in understanding the functional redundancy and hierarchical environmental controls on stream microbial structure and function (Goodman et al., 2015).

### Lotic Microbial Diversity is Critical and Understudied

While the importance of microbial diversity to ecosystem function, and the threats that environmental changes pose to diversity and function, are key areas of research in all aquatic ecosystems, lotic microbial diversity has generally received less attention than marine and lentic microbial diversity (Zinger et al., 2012). Comparing and contrasting key points in the current state of knowledge in both lotic and better-studied aquatic systems highlights areas where complementary research can further advance understanding of diversity-function relationships in aquatic systems generally.

While most work on aquatic microbial diversity has focused on planktonic communities (Zwart et al., 2002; Hahn, 2006; Smith, 2007; Newton et al., 2011; Ladau et al., 2013), in lotic ecosystems the diversity of surface-attached biofilms in benthic habitats is better studied (**Figure 1D**). The dominant controls on bacterioplankton diversity, such as light and algal abundance, predation, temperature and salinity (Fuhrman et al., 2006; Kent et al., 2007; Lefort and Gasol, 2013), may differ from dominant controls on benthic microbial diversity, due to the contrasting energy sources and physical conditions of pelagic versus benthic habitats (Covich et al., 2004). A predominant contrast between these habitats is the relative importance of algal vs. terrestrial-derived OM substrates, and the coarse differences between water column, epilithon, CPOM, FBOM, and sediment associated bacterial community composition in lotic systems (**Figure 4**; **Table 2**) may be related in part to autochthonous versus allochtonous OM source, as noted earlier. While it is possible that the structure and function of microbiota on allochthonous substrates responds differently to environmental changes than those associated with autochthonous substrates (Dodds, 2006), like primary production, decomposition and microbial mineralization of terrestrial OM in streams responds positively to changes in nutrient concentrations and temperature (Boyero et al., 2011; Rosemond et al., 2015). The topic of microbial metabolism of algal versus terrestrial OM is increasingly relevant in all aquatic systems (Del Giorgio et al., 1997; Pace et al., 2004), and hypotheses based on microbial diversity and function in heterogeneous lotic habitats can inform understanding of carbon cycling across changing, and connected, aquatic landscapes (Battin et al., 2008).

In addition to the organic substrate heterogeneity presented by the benthos-dominated lotic ecosystem, the level of interaction between benthic and pelagic habitats affects aquatic diversity and function (Covich et al., 2004). Bacterioplankton community composition can be temporally predictable, in concert with temporal variation in environmental parameters such as light and temperature (Fuhrman et al., 2006; Kan et al., 2006; Kent et al., 2007; Crump et al., 2009; Eiler et al., 2012). Benthic surface- and sediment-attached microbiota are exposed to variability in physical factors, such as shear stress and surface roughness, as well as changes in light and water quality, and biofilm formation is a predominant characteristic of lotic microbiota. Thus, the large heterogeneity in surfaces available for colonization within a stream reach presents a variety of contrasting selective environments (Battin et al., 2007). Also, bacterioplankon diversity is impacted by water residence time, (Crump et al., 2004; Lindström and Bergström, 2004), and the flowing water of lotic systems clearly acts to transport microbial cells relatively quickly from up- to downstream habitats (Crump et al., 2007; Riemann et al., 2008). Thus, both dispersal and environmental filtering are strong forces in microbial biofilm community assembly in stream and river ecosystems, making lotic systems highly appropriate for understanding metacommunity dynamics (Logue et al., 2011; Besemer et al., 2012, 2013).

Surface-attached and bed sediment-entrained cells are not always accounted for in broad views of microbial life in aquatic ecosystems (Whitman et al., 1998), yet their activity drives biogeochemical processes at reach, watershed and continental scales (Likens et al., 1970; Mulholland et al., 2008; Rosemond et al., 2015). Further research on lotic microbial diversity, including themes such as community assembly, OM source and metabolism, and functional diversity and redundancy under multi-factor environmental variability, is critical to understanding and managing aquatic ecosystem functions in a changing world. Yet lotic microbial diversity is still understudied: Many studies to date are observational, few are experimental; most take place within one stream, few employ cross-site comparisons that would facilitate identification of universal drivers. Given the importance of stream microbes to global biogeochemical cycles, the rapidly increasing accessibility of molecular tools and data, and the relevance of stream microbiota to larger ecological questions, there is still a lot to be learned about lotic microbial diversity.

## Author Contribution

LZ collected and synthesized the data and wrote the manuscript.

## Conflict of Interest Statement

The author declares that the research was conducted in the absence of any commercial or financial relationships that could be construed as a potential conflict of interest.
